# The acute inflammatory response to copper(II)-doped biphasic calcium phosphates

**DOI:** 10.1016/j.mtbio.2023.100814

**Published:** 2023-10-04

**Authors:** L. Thoraval, E. Thiébault, R. Siboni, A. Moniot, C. Guillaume, A. Jacobs, J.-M. Nedelec, G. Renaudin, S. Descamps, O. Valfort, S.C. Gangloff, J. Braux, D. Marchat, F. Velard

**Affiliations:** a Université de Reims Champagne-Ardenne, EA4691 “Biomatériaux et Inflammation en site osseux” BIOS, Reims, France; b Université Clermont Auvergne, CNRS, Clermont Auvergne INP, ICCF, Clermont-Ferrand, France; c Mines Saint-Etienne, Univ Lyon, CNRS, UMR 5307 LGF, Centre SPIN, F-42023, Saint-Etienne, France; d Mines Saint-Etienne, Univ Jean Monnet, Etablissement Français du Sang, INSERM, U 1059 Sainbiose, 42023, Saint-Etienne, France

**Keywords:** Biomaterials, Cu-doped biphasic calcium phosphate, Cytokines, NETosis, Inflammation

## Abstract

Infection and inflammation are two key features to consider to avoid septic or aseptic loosening of bone-implanted biomaterials. In this context, various approaches to fine-tune the biomaterial's properties have been studied in order to modulate the crosstalk between immune and skeletal cells. Cation-doping strategies for tuning of calcium phosphates properties has been evidenced as a promising way to control the biomaterial-induced inflammatory process, and thus improving their osteoimmunomodulatory properties. Copper(II) ions are recognized for their antibacterial potential, but the literature on their impact on particulate material-induced acute inflammation is scarce. We synthesized copper(II) ions-doped biphasic calcium phosphate (BCP), intended to exhibit osteoimmunomodulatory properties. We addressed *in vitro*, for the first time, the inflammatory response of human primary polymorphonuclear neutrophils (PMNs) to copper(II) ions-doped or undoped (BCP) powders, synthesized by an original and robust wet method, in the presence or absence of LPS as a costimulant to mimic an infectious environment. ELISA and zymography allowed us to evidence, *in vitro*, a specific increase in IL-8 and GRO-α secretion but not MIP-1β, TNF-α, or MMP-9, by PMNs. To assess *in vivo* relevance of these findings, we used a mouse air pouch model. Thanks to flow cytometry analysis, we highlighted an increased PMN recruitment with the copper(II) ions-doped samples compared to undoped samples. The immunomodulatory effect of copper(II) ions-doped BCP powders and the consequent induced moderate level of inflammation may promote bacterial clearance by PMNs in addition to the antimicrobial potential of the material. Copper(II) doping provides new insights into calcium phosphate (CaP)-based biomaterials for prosthesis coating or bone reconstruction by effectively modulating the inflammatory environment.

## Highlights

• Copper(II) ions doping potentiates neutrophil recruitment *in vivo* in a murine air pouch model.
• Copper(II) ions doping maintains a moderate level of inflammation in addition to providing antibacterial potential.
• Copper(II) ions doping does not induce an exaggerated proteolytic environment.
• Copper(II) ions doping provides new insights into calcium phosphate (CaP)-based bone replacement and regeneration.
• Copper(II) ions-doped biphasic calcium phosphate powder synthesis is achievable at an industrial GMP scale.

## Graphical abstract

**Figure:**
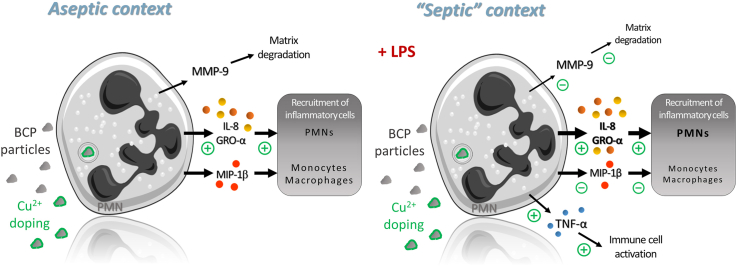

## 1 Introduction

Calcium phosphate (CaP)-based biomaterials, especially hydroxyapatite (HA, Ca_10_(PO_4_)_6_(OH)_2_) and β-tricalcium phosphate (β-TCP, Ca_3_(PO_4_)_2_), are widely used as medical devices for the restoration and treatment of bone tissue defects or as coatings for prosthesis integration, mainly because they induce intimate and functional interfaces with the host bone [1,2], and their degradation products are naturally metabolized [3]. Despite their good biocompatibility and osteoconductivity [4], they have a limited ability to promote osteogenesis and angiogenesis, to modulate the crosstalk between the immune and bone cells [5], and suffer from inappropriate biodegradability, which globally impedes bone regeneration [6]. A mixture of HA and β-TCP, called biphasic calcium phosphate (BCP), is usually preferred over pure HA or β-TCP to balance the resorption rate for adaptation to clinical needs [7], as well as to generate a more favorable osteoimmunomodulatory microenvironment [8]. In this way, BCP meets the requirements of an ideal bone substitute quite well; nevertheless, progress remains to be made in controlling the inflammatory and infectious risks related to implantation. First, CaP-based biomaterials have a tendency to fragment and generate particle wear debris due to the movements taking place at the prosthesis interface (i.e., surgical placement and joint movements), which can lead to aseptic implant loosening [9]. It has been demonstrated that CaP particles have the capacity to recruit and activate inflammatory cells, including polymorphonuclear neutrophils (PMNs) and monocytes/macrophages, and produce inflammatory mediators such as proteases and cytokines/chemokines [[10], [11], [12]]. This inflammatory response, if uncontrolled, disturbs the bone balance toward bone resorption and could result in implant loss [13].

Second, the risk of bacterial infection is another issue that needs to be considered during the implantation of a bone substitute, especially because infections in bone sites are complicated to treat due to their deep localization and poor vascularity in the tissue. Infection rates vary from 0.7 to 4.2% in elective orthopedic surgery and up to 30% in third degree open bone fractures [14]. Bone infections have serious consequences, including delayed healing, the need for additional surgeries and a longer hospitalization time and consequently increased costs [15]. Natural bone calcium-deficient apatite accommodates many ions from blood plasma in its structure (principally Na^+^, K^+^, Mg^2+^, Cl^−^, and HCO_3_^−^), and apatite can undergo various substitutions, including from carbon, modifying the physicochemical and metabolic properties of the bone [16]. To control the inflammatory response, studies have focused on chemically modifying CaP-based bioceramics by means of cationic dopants including zinc and strontium [17,18]. On the same way metal ions have been incorporated into BCP materials to control bacterial growth such as selenium [19], manganese [20], magnesium [21], or copper [22].

On the one hand, copper is an essential trace element involved in various biological processes and has recently been thrust into the spotlight for its antibacterial properties [22,23]. Studies have demonstrated that copper(II) ions can produce reactive oxygen species (ROS) and cause damage to lipids, proteins and DNA, leading to the death of microorganisms that come in contact with copper(II) ions-doped CaP materials [24,25]. On the other hand, it has also been shown that copper(II) ions improve the angiogenic and osteogenic potential of materials [26,27]. Since adding a copper(II) coating on titanium implants has been shown to have proinflammatory potential [28], it was also suggested that the local inflammatory response induced by the release of copper(II) ions could be controlled by the biomaterials themselves [29]. Moreover, the incorporation of copper(II) ions into biomaterials could endow the material with favorable inflammation-modulating properties to activate macrophages for enhanced osteogenesis and bacterial killing effects [30]. Thus, copper(II) ions appear to be a promising doping element to improve the abilities of CaP in bone repair surgery [31].

In the present work, we investigated the impact of copper(II) ions doping of BCP on the acute inflammatory response. Assays were performed with copper(II) ions-doped BCP powders synthesized by an original wet method based on the precipitation and the hydrolysis of copper(II)-doped brushite. The first objective of this work was to propose a highly stable and reproducible process for the production of copper(II)-doped BCP powders that would comply with Good Manufacturing Practice (GMP), while limiting the environmental impact of such production. To achieve this, the obvious principles of ionic speciation were applied, particularly for copper(II) ions. For the first time a reaction mechanism (i.e., set of elementary reactions) for the precipitation of copper-doped deficient hydroxyapatites was proposed and confronted with physico-chemical analyses. The second objective of this work was to emphasize copper(II) ions immunomodulatory effect on PMN activation. For the first time using human primary neutrophils, we characterized their production of the chemokines interleukin-8 (IL-8), GRO-α, macrophage inflammatory protein-1β (MIP-1β) and the proinflammatory mediator tumor necrosis factor-α (TNF-α). The gelatinolytic activity of matrix metalloproteinase-9 (MMP-9), and the release of neutrophil extracellular traps (NETs) were also investigated *in vitro*. To gain insight on *in vivo* relevance of copper(II)-doping impact on PMN recruitment, the murine ai pouch model and flow cytometry were used to evaluate cell infiltrates. The levels of the murine IL-8 homolog KC and TNF-α were measured in air pouch exudates. These original approaches coupled together shed light on the first evidence to date of immunomodulatory effect of copper(II)-doped BCP intended to be used in bone.

## 2 Materials and methods

### 2.1 Media and reagents

Hexamethyldisilazane (HMDS), 0.22 μm filters, ethylene diamine tetracetic acid (EDTA), tris(hydroxymethyl)aminomethane (TRIS), sodium dodecyl sulfate (SDS), paraformaldehyde, glutaraldehyde, penicillin‒streptomycin, dimethylsulfoxide (DMSO), lipopolysaccharide from *Escherichia coli* 0111: B4 (LPS), glycin, gelatin from bovine skin, acrylamide/bisacrylamide 30% solution, recombinant human pro-MMP-9 and the cytotoxicity detection kit were purchased from Sigma Aldrich (Saint-Quentin Fallavier, France). Polymorphprep™ was purchased from ProteoGenix (Schiltigheim, France). Ethanol was purchased from Charbonneaux-Brabant (Reims, France). Coomassie Blue G-250 was purchased from Bio-Rad (Marnes-la-coquette, France). Roswell Park Memorial Institute (RPMI) 1640 medium, Dulbecco's phosphate-buffered saline (DPBS), streptavidin-FITC, 4′,6-diamidino-2-phenylindole (DAPI), and paraffin Shandon™ were purchased from ThermoFisher Scientific (Illkirch-Graffenstaden, France). Triton X100, sodium chloride, calcium chloride, acetic acid, xylene, and methanol were purchased from VWR (Strasbourg, France). Isoflurane IsoFlo® was purchased from Zoetis (Malakoff, France). The ELISA DuoSet kits for human TNF-α and IL-8 were purchased from R&D systems (Biotechne, Rennes, France). Syringes (10 mL) and 27G needles were from Terumo®Agani™. Fluorescent mounting medium was obtained from Agilent-DAKO. Recombinant rabbit anti-mouse Ly6G (ER + PR22909-135) was purchased from Abcam (Cambridge, UK). Goat anti-rabbit IgG biotinylated antibody and Bloxall® Endogenous Blocking Solution were purchased from Vector Laboratories (Burlingame, CA, USA).

### 2.2 Generation of the Cu-doped BCP samples

BCP powders were prepared *via* an aqueous precipitation method [32] using a fully automated apparatus composed of jacketed reactors (20 and 30 L, De Dietrich, France), cryothermostats (Huber, Germany), stirring devices, and peristaltic (Masterflex L/S, U.S.A.) and dosing (ProMinent, UK) pumps. These powders were named “AP” for aqueous precipitation. Undoped calcium-deficient hydroxyapatite (CDHA) was obtained by a conventional method where a diammonium hydrogen phosphate aqueous solution ((NH_4_)_2_HPO_4_, EMSURE® ACS, Merck, Germany, [P] = 1.43 mol/L) was added at a rate of 350 mL/min to a calcium nitrate solution (Ca(NO_3_)_2_·4H_2_O, 99% pure, Merck, Germany, [Ca] = 2.33 mol/L) with stirring (600 rpm). The reaction was performed under an argon flow (4.8, Air Products, 0.1 mL/min) to prevent the formation of carbonate-containing apatite. The pH of the suspension was adjusted to 7.0 by the addition of 28% ammonia solution (Merck, 28–30% EMSURE® ACS, Germany) using a dosing pump coupled with a pH controller (Mettler Toledo M400, U.S.A.) and a pH electrode (Mettler Toledo Inpro 4800/120/PT100, U.S.A.). The temperature was controlled and regulated automatically at 35 °C with an external T-probe connected to a cryothermostat. After complete introduction of the phosphate solution, the suspension matured for 19 h and was finally centrifuged at 4000 rpm for 5 min (Thermo Fisher Scientific, Sorvall Legend XF, France). Based on original unpublished data and to obtain a CDHA powder with a final Ca/P molar ratio of 1.620 (i.e., Ca_10-*x*_(HPO_4_)_*x*_(PO_4_)_*x*_(OH)_2-*x*_ with *x* = 0.28), the Ca/P molar ratio of the reagents was fixed at 1.680 (cf. Table 1 for complementary data) under these synthesis conditions (sc. kinetic control). The general precipitation reaction in aqueous solution can be written as follows (Eq. (1)).

Eq. 1 $$\left(10-x\right)\;{\text{Ca}}^{2+}+6\;{\text{HPO}}_{4}^{2-}+\left(8-2x\right)\;{\text{OH}}^{-}\;⇆\;{\text{Ca}}_{10-x}{\left({\text{PO}}_{4}\right)}_{6-x}{\left({\text{HPO}}_{4}\right)}_{x}{\left(\text{OH}\right)}_{2-x}+\left(6-x\right)\;H_{2}O$$

with 0≤x ≤ 1.

**Table 1:** Parameters of the synthesis of the aqueous precipitation-based powders, their final compositions after heat treatment at 1050 °C for 5 h under 93 kPa of N_2_ and 7 kPa of H_2_O, and the Ca, Cu and P concentrations in RPMI 1640 Glutamax® medium incubated for 4 h at 37 °C with the Cu_z_BCP powders (1.7 mg/mL).

Sample name	Chemical synthesis: Reagent amounts				Final calcined powder composition				Powder dissolution: Element concentration in RPMI media		
	z	n_Ca_	n_P_	n_Cu_	HA/β-TCP mass ratio	Ca/P	x	Cu/Ca^b^	Ca	Cu	P
	–	/ mol	/ mol	/ mol	From Rietveld and [33]	from Eq. 4	Eq. 4	by ICP/AES	/ mol/L	/ mol/L	/ mol/L
RPMI media	–	–	–	–	–	–	–	–	0.62 ±0.02	0.00 ±0.00	5.25 ±0.18
Cu_0_BCP	0	23.29	13.86	0	76 ± 1/24 ± 1	1.624 ±0.001	0.26	0	0.63 ±0.02	0.00 ±0.00	5.34 ±0.05
Cu_0.1_BCP	0.1	11.87	7.12	0.119	63 ± 1/37 ± 1	1.601 ±0.001	0.39	0.011 ±0.002	0.63 ±0.01	0.06 ±0.01	5.29 ±0.08
Cu_0.2_BCP	0.2	11.80	7.08	0.236	75 ± 1/25 ± 1	1.623 ±0.001	0.26	0.019 ±0.003	0.62 ±0.02	0.28 ±0.01	5.24 ±0.06

Cu-doped calcium-deficient hydroxyapatite (CDCuHA) was prepared through two successive reactions: precipitation and then hydrolysis of copper(II) ions -doped brushite. This method was chosen because copper(II) ions are stable below pH 5–6 [34,35], brushite (dicalcium phosphate dihydrate, DCPD, CaHPO_4_·2H_2_O) is the most stable phase of the calcium phosphate system in acidic environments [33], and a Ca_1-x_Cu_x_HPO_4_ solid solution with 0≤x < 0.25 exists [34]. The first stage of the process consisted of adding diammonium hydrogen phosphate aqueous solution (EMSURE® ACS, Merck, Germany, [P] = 0.80 mol/L) to a homogeneous mixture of calcium and copper(II) nitrate solutions (Ca(NO_3_)_2_·4H_2_O, Cu(NO_3_)_2_·3H_2_O, EMSURE®, Merck, Germany, [Ca] = 1.08 mol/L, [Cu] = 0.01 mol/L) at a rate of 100 mL/min to form a copper(II)-doped brushite (Ca_(1-z1)_Cu_(z1)_HPO_4_·2H_2_O) [36] according to the following general reaction scheme (3.5 < pH < 4.5).

Eq. 2 $$\left(1-z_{1}\right)\;{\text{Ca}}^{2+}+z_{1}\;{\text{Cu}}^{2+}+H_{2}{\text{PO}}_{4}^{2-}\;⇆\;{\text{Ca}}_{\left(1-z1\right)}{\text{Cu}}_{\left(z1\right)}{\text{HPO}}_{4}\cdot 2H_{2}O+H_{3}O^{+}$$

with z_1_ < 0.25.

Precipitation was performed with an excess of cations with respect to the stoichiometry of the reaction in Eq. (2) (Ca/P = 1.667 and (Ca + Cu)/P = 1.683 or (Ca + Cu)/P = 1.700, Table 1), and the pH was not adjusted and varied between 3.5 and 4.5. This suspension was maintained with stirring (550 rpm) under an argon flow for 3 h at 40 °C. The conversion of copper(II)-doped brushite into CDCuHA was made by adjusting the reaction pH at 7.0 with 28% ammonia solution according to the general reaction (Eq. (3)) considering the interstitial insertion of copper(II) ions into the HA crystal lattice (Wyckoff site 2*b*) [31].

Eq. 3 $$\left(4-x+6z_{1}\right)\;{\text{Ca}}^{2+}+z_{2}\;{\text{Cu}}^{2+}+6\;{\text{Ca}}_{\left(1-z1\right)}{\text{Cu}}_{\left(z1\right)}{\text{HPO}}_{4}\cdot 2H_{2}O+\left(8-2x+2z\right)\;{\text{OH}}^{-}\;⇆\;{\text{Ca}}_{10-x}{\text{Cu}}_{z}{\left({\text{PO}}_{4}\right)}_{6-x}{\left({\text{HPO}}_{4}\right)}_{x}{\left(\text{OH}\right)}_{2-2z-x}{\left(O\right)}_{2z}+\left(6-x+2z\right)\;H_{2}O$$

with z = 6z_1_ + z_2_, 0 ≤ x ≤ 1, and z ≤ 0.2.

The reaction continued for 24 h at 40 °C with stirring (550 rpm) with nitrogen gas bubbling and then stopped by centrifugation of the suspension at 4000 rpm for 5 min.

Three compositions of CDCu_z_HA (Ca_10-x_Cu_z_(PO_4_)_6-x_(HPO_4_)_x_(OH)_2-2z-x_(O)_2z_) were prepared with z = 0, 0.1 and 0.2, corresponding to 0%, 0.6% and 1.3% w/w Cu with respect to the CDCu_z_HA molar mass (Table 1). Immediately after centrifugation, both the CDHA and CDCuHA wet cakes were frozen at −80 °C, then freeze-dried (Pilote Compact, Cryotec, France) and finally sieved with ethanol absolute (VWR, France) on 11 μm nylon sieve (Fisher Scientific, France) to obtain the raw powders. Subsequently, CDHA and CDCuHA raw powders were heat treated at 1050 °C for 5 h under 93 kPa of N_2_ and 7 kPa of H_2_O (ramped at 4 °C/min) and again sieved with ethanol absolute (VWR, France) on 25 μm nylon sieve (Fisher Scientific, France). The gas mixture N_2_/H_2_0 was provided by a controlled humidity generator (WETSYS 0–200 mL/min, Setaram Instrumentation, France) programmed to deliver a gas at 40 °C with 90% relative humidity and a flow rate of 150 mL/min. During these heat treatments, CDHA and CDCuHA decomposed into undoped (BCP) and Cu-doped (Cu_z_BCP, with z being the stoichiometric factor in CDCuHA in Eq. (3)) biphasic calcium phosphates according to the following general reaction occurring between 720 °C and 840 °C.

Eq. 4 $${Ca}_{10-x}Cu_{z}{\left({PO}_{4}\right)}_{6-x}{\left({HPO}_{4}\right)}_{x}{\left(\text{OH}\right)}_{2-2z-x}{\left(O\right)}_{2z}\;\overset{\Delta }{\to }\;\left(\frac{1-x+z}{1+z}\right)\;{Ca}_{10}Cu_{z}{\left({PO}_{4}\right)}_{6}{\left(\text{OH}\right)}_{2-2z}{\left(O\right)}_{2z}+\left(\frac{3x}{1+z}\right)\;{Ca}_{3-}\frac{z}{3}Cu\frac{z}{3}{\left({PO}_{4}\right)}_{2}+\left(\frac{x\;\left(1-z\right)}{1+z}\right)\;H_{2}O$$

with 0 ≤ *x* ≤ 1 and *z* ≤ 0.2

The BCP powders previously synthesized *via* the sol-gel route, heat treated for 15 h at 900 °C under air and characterized by Gomes et al. [31] were used to confirm the proof-of-concept copper(II) ions-doping effect independent of the synthesis route; the results obtained with these sol-gel-based BCP powders are provided in the supplementary material.

Before the biological assays, the powders were sterilized by dry heat in a Poupinel oven (MEMMERT) for 2 h at 250 °C and then suspended in RPMI 1640 Glutamax® medium immediately before use.

### 2.3 Characterization of the Cu-doped BCP samples

Diffractograms were collected at room temperature with a D8 A25 θ/θ diffractometer (Bruker AXS, Germany) equipped with a Lynx-Eye_XE_T Detector (aperture angle ≈3°) using CuKα radiation (λ = 1.5418 Å) and operating at 40 kV and 40 mA. The X-ray diffraction (XRD) patterns were collected over the 2θ range of 10–100° with a step size of 0.01° and counting time of 0.3 s per step. *TOPAS-64 v6 software* (Bruker AXS, Germany) *was used* for qualitative (peak fitting) and quantitative (Rietveld refinement) phase *analysis* and *crystal structure refinement* (Pawley and Le Bail methods) [37]. The quantitative HA/β-TCP mass ratio obtained from *Rietveld structure refinement was also compared to that from* a standard procedure based on XRD calibration curves [38]. The crystalline phases were identified after comparison with reference patterns from the ICDD-PDF database (International Centre for Diffraction Data –Powder Diffraction Files). Refinements were performed using the space groups of the HA hexagonal structure P63/m (176, PDF 00-009-0432) and the β-TCP trigonal structure R3c (167, PDF 00-009-0169). The initial cell parameters were taken as *a* = 9.418 Å and *c* = 6.884 Å and *a* = 10.429 Å and *c* = 37.380 Å, respectively.

Powders were characterized by Fourier transform infrared (FTIR) spectroscopy using a VERTEX 70 spectrometer (Bruker Optics, France) equipped with a monolithic diamond ATR crystal (Quest ATR diamond; Specac, USA). Spectra were recorded from 4000 to 400 cm^−1^ at a resolution of 2 cm^−1^ and obtained by averaging the signals from 64 successive scans.

The phosphorous, calcium and copper contents in the powders and RPMI 1640 Glutamax® culture medium were determined by means of inductively coupled plasma atomic emission spectroscopy (ICP/AES, Horiba Activa Spectrometer, Jobin-Yvon). Powder samples were dissolved in 1 M nitric acid solution (EMSURE® ACS, Merck, Germany) in such a manner to limit measurement uncertainty and be within the highest sensitivity range of the ICP/AES instrument (range 1–100 ppm). To determine the amount of copper(II) ions released by Cu_z_BCP powders into RPMI 1640 Glutamax® medium containing 1% penicillin/streptomycin and 2.5% heat-inactivated autologous human serum, 1.7 mg of each powder was incubated for 4 h at 37 °C in 1 mL of culture medium in a 24-well cell culture plate. The powder suspensions were centrifuged at 13 000 g for 10 min, and the supernatants from 12 wells were pooled and then filtered through a 0.22 μm filter. The obtained solutions were finally mixed with 1 M nitric acid solution at a 2:1 vol ratio, filtered again (0.22 μm) and analyzed.

The specific surface area (SSA) of each sample was measured twice on each powder outgassed at 200 °C for 8 h by means of the Brunauer−Emmett−Teller (BET) five-point method using N_2_ adsorption isotherms (Micromeritics ASAP 2010, Germany).

### 2.4 Collection of blood samples and cell isolation and culture

Venous blood was collected on EDTA from healthy donors of the “Etablissement Français du Sang Grand Est” (Authorization ALC/PIL/DIR/AJR/FO/606 Reims, France) after written informed consent was obtained from the donors. PMNs were isolated from whole blood collected on EDTA (Vacutainer K2E tubes, Becton Dickinson) using the Polymorphprep™ protocol. Residual erythrocytes were removed by hypotonic shock. Resulting neutrophils were resuspended in RPMI 1640 Glutamax® medium with 1% penicillin/streptomycin and 2.5% heat-inactivated autologous human serum and were counted with a Neubauer chamber (Hirschmann®, Eberstadt, Germany). Neutrophils were cultured in contact with each BCP powder previously suspended at the desired concentration in RPMI medium. Two negative controls, one with cells without material and the other containing culture medium alone, were systematically performed to indicate the initial inflammatory state of the donor. A positive control of inflammation, containing material-free cells stimulated with 10 ng/mL LPS, was also performed.

PMN/BCP powder cultures were carried out in 24-well plates with one million cells in 1 mL at a constant powder mass (1.7 mg/mL) [28] with or without costimulation using LPS (10 ng/mL). For imaging, cells and powders were seeded onto Thermanox™ coverslips (ThermoFisher Scientific). Unless otherwise stated, after 4 h of incubation at 37 °C in a humidified atmosphere with 5% CO_2_, the contents of the wells were collected. After centrifugation (600 g, 20 °C, 10 min), the cells were removed, and the clarified supernatants (6000 g, 20 °C, 10 min) were frozen at −20 °C for further analysis.

### 2.5 ELISA

The IL-8, GRO-α, MIP-1β, and TNF-α concentrations in the PMN-conditioned supernatants and murine KC and TNF-α in the air pouch exudates were measured using an enzyme-linked immunosorbent assay (ELISA) kit following the manufacturer's instructions. Protein concentrations were estimated using a standard concentration curve of human recombinant IL-8, GRO-α, MIP-1β, or TNF-α, and mouse recombinant KC or TNF-α. Controls included nonstimulated cells and medium alone. Absorbance measurements were performed at 450 nm corrected at 570 nm using a microplate reader FLUOstar Optima® (BMG Labtech).

### 2.6 Gelatin zymography

Gelatin zymography was used to detect pro-matrix metalloproteinase-9 (pro-MMP-9)-related gelatinolytic activity in PMN-conditioned supernatants. The total protein content in the supernatants was determined by measuring the absorbance at 280 nm with a Nanodrop™ spectrophotometer (ThermoFisher Scientific), and equal amounts (2 μg) were used for zymography experiments. The negative control supernatants (cells alone and culture medium alone) and positive control supernatants (cells stimulated with LPS) were systematically deposited on each gel. A control lane containing 20 μL of recombinant human pro-MMP-9 (final concentration, 50 μL/mL) was also performed. The gelatinolytic activities in the supernatants were evaluated with 10% SDS-polyacrylamide gels containing 1 mg/mL gelatin. Migration was carried out in buffer (TRIS, SDS and glycin) for 30 min at 80 V in the compression gel and then at 160 V in the separation gel using a Bio-Rad Power Pac 200. After electrophoresis, SDS was removed from the gel by two incubations in 2.5% Triton X100 for 30 min at room temperature. Then, the gels were incubated for 18 h at 37 °C in 50 mM Tris-HCl (pH 7.6), 0.2 M NaCl and 5 mM CaCl_2_. The gels were stained for 30 min with Coomassie Blue G-250 and then destained (2 × 30 min) in 10% acetic acid and 20% methanol. Gel digitalization was performed on a Vilber Lourmat CN-UV/WL system controlled by BioCapt® software. Proteolytic activity was detected as clear bands against the blue background of stained gelatin and quantified as integrated optical density (IOD) by ImageJ software. Three forms of pro-MMP-9 were detected: pro-MMP-9 (92 kDa), pro-MMP-9 complexed with lipocalin (N. GAL 135 kDa) and pro-MMP-9 homodimers (235 kDa) [39].

### 2.7 NET observation and quantification

Scanning electron microscopy (SEM) was utilized to observe the formation of NETs by PMNs in contact with the powders. After 4 h of culture with or without LPS costimulation, the supernatants were removed, and cells were fixed with 2.5% glutaraldehyde in 1X DPBS for 1 h at room temperature. After two washes with distilled water for 10 min each time, the cells were dehydrated in graded ethanol solutions (50%, 70%, 90% and 100% two times) for 10 min. Samples were finally desiccated with HMDS for 5 min. The samples were then sputtered with a thin gold-palladium film using a JEOL ION SPUTTER JFC-1100 instrument (8 mA and 1200 V, 8 min). Cells were observed using a Schottky field emission scanning electron microscope (JEOL JSM-7900F). The soluble nucleic acids were quantified in the supernatants using the *Quant-It* ^*TM*^ *dsDNA High-Sensitivity* kit (ThermoFisher, Scientific) following the manufacturer's instructions. Fluorescence was measured at ex485/em520 nm using a microplate reader (FLUOstar Optima®, BMG Labtech).

### 2.8 LDH measurement

Lactate dehydrogenase (LDH) activity was evaluated in neutrophil-conditioned media using a cytotoxicity detection kit following the manufacturer's instructions (Sigma-Aldrich). Absorbance was measured at 490 nm with correction at 700 nm using a microplate reader (FLUOstar Optima®).

### 2.9 Air pouch model

These experiments were performed on mice housed in a controlled environment (temperature: 21 ± 2 °C, relative humidity: 65 ± 15%, and natural alternating light/dark cycle (agreement no B514543) in accordance with UE Directive 2010/63/EU and protocols approved by the Regional Ethics Committee on Animal Experimentation (CEEA no 056) and the Ministry of Agriculture under the direction of investigators certified for animal experiments following protocol APAFIS# 13375–2017121218136235. Seven-week-old male BALB/c mice were acclimated for 1 week before air pouch induction. Before each injection, mice were anesthetized using 5% isoflurane. On day 0, we filtered air through a 0.22 μm filter. Three milliliters of filtered air were subcutaneously injected with a 10 mL syringe and 27 G needle in the middle of the back of each mouse. On day 3, the injection was repeated. On day 7, inflammation was induced by injecting 2 mL of LPS (10 ng/mL) or 2 mL of BCP powder (5 mg/mL) resuspended in DPBS. Control mice (sham condition) were injected with 2 mL of DPBS 0.5% DMSO. After 6 h, intracardiac blood was collected under gaseous anesthesia before euthanasia of the animals. To remove the exudate from the air pouch, 2 mL of DPBS-EDTA (5 mM) was injected, and after 30 s of soft massage of the pouch, exudate was collected. Cells from the exudate were washed and counted using a Kova® slide (Dutsher, Brumath, France).

### 2.10 Flow cytometry

To evaluate leukocyte subsets and discriminate monocytes and macrophages, one million cells were collected from each air pouch sample and then stained with the fluorescent antibodies anti-Ly6G and anti-F4/80. After washing, the cells were fixed in BD Lyse/Fix and used for flow cytometry analysis with a BD LSRFortessa™ system. F4/80-BV421 was excited with a 405 nm laser and detected with an emission filter of 450/50 nm. Ly6G-PE was excited with a 561 nm laser and detected with an emission filter of 585/15 nm. At the first stage, single cells were selected by an FSC-H/FSC-A gate. In this population, cells were selected by an FSC-A/SSC-A gate excluding subcellular debris. Then, PMNs were selected by a Ly6G-PE + gate on an SSC-A/Ly6G-PE dot plot. Macrophages and monocytes were discriminated on an SSC-A/F4/80-BV421 dot plot. Data analysis was performed with FlowLogic software (Miltenyi Biotec).

### 2.11 Histological analysis

For histological analysis, the collected air pouches were fixed in formaldehyde 4% for one week, washed in an increasing series of alcohols, followed by clearing in xylene and embedding in paraffin. Multiple serial sections were made at a thickness of 5 μm. Sections were rehydrated and stained with Masson's trichrome (hematoxylin, fuchsin and aniline blue). Immunohistochemical analysis was carried out on following sections to evaluate the neutrophil population. Samples were blocked with Bloxall® Endogenous Blocking Solution and stained using a primary anti-Ly6G antibody (1:4000 in 1% BSA) and a secondary goat anti-rabbit antibody (1:50 in 1% bovine serum albumin (BSA)). Streptavidin-FITC was then added (1:2000 in 1% BSA), and nuclear staining was performed with DAPI (1:3000 in PBS). Sections were then mounted with Dako Fluorescent Mounting Medium and a coverslip, and observations were made using a Zeiss Axiovert 200 M inverted microscope coupled with AxioVision™ v2.8 software.

### 2.12 Statistical analysis

Each *in vitro* experiment was performed on cells from 9 to 11 independent human donors, and *in vivo* tests included 9–16 mice. The significance of the results was first assessed with the nonparametric Kruskal‒Wallis test followed by *post hoc*, exact (stratified for blood donors) nonparametric Wilcoxon Mann‒Whitney test (StatXact 7.0, Cytel Inc, MA, USA). We used nonparametric statistics owing to the lack of a normal distribution of the assessed variables. Stratification allowed the impact of donor variability to be considered for *in vitro* studies. Differences were considered significant at *p* < 0.05. Unless otherwise stated, the red bar represents the median value, black bars represent the 1^st^ and 9^th^ deciles, the limits of rectangles represent the 1^st^ and 3^rd^ quartiles, and triangles represent the average values.

## 3 Results

### 3.1 BCP sample characterization

**Fig. 1:**
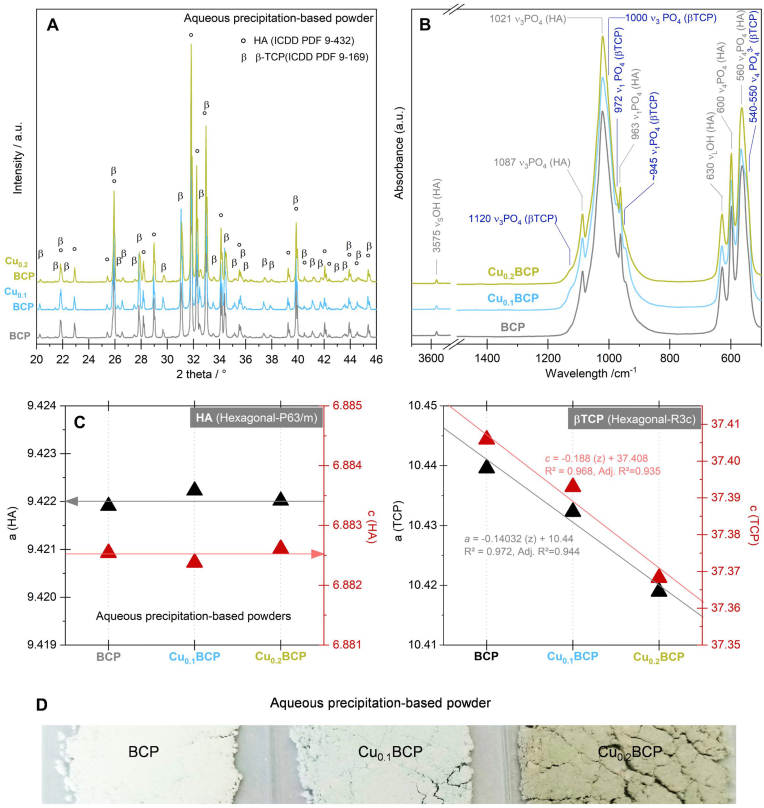
Physicochemical characterization of aqueous precipitation-based powders. (**A**) XRD patterns, (**B**) IR spectra, (**C**) lattice parameters (*a* and *c*), and (**D**) colors of the BCP, Cu_0.1_BCP and Cu_0.2_BCP powders heat treated at 1050 °C for 2 h under 93 kPa of N_2_ and 7 kPa of H_2_O. *The hexagonal* structure, *P63*/*m* (*176*) space group, according to ICCP-PDF 9–432, and *trigonal* structure, *R3c* (*167*) space group, according to ICCP-PDF 9–169, were used for XRD phase identification. (For interpretation of the references to colour in this figure legend, the reader is referred to the Web version of this article.)

Both the diffractograms (Fig. 1A) and FTIR spectra (Fig. 1B) of the powders heat treated at 1050 °C for 5 h under 93 kPa of N_2_ and 7 kPa of H_2_O exhibited the characteristic diffraction lines or bands of the HA and β-TCP phases (PDF 00-009-432 and 00-009-169, respectively). No other crystalline or amorphous phases were detected. The percentages by mass of the HA and β-TCP crystalline phases were assessed to be on the same order of magnitude for all Cu_z_BCP samples, i.e., z = 0, 0.1 and 0.2 (Table 1), with equivalent results between the Rietveld refinement procedure and standard calibration curve method. Moreover, crystal structure refinement showed that the hexagonal lattice parameters *a* and *c* of the samples did not change with the amount of copper(II) (z). Conversely, both *a* and *c* of the trigonal structure decreased linearly with increasing z (Fig. 1C). The color of the heat-treated samples was dependent on the copper(II) doping values: white for BCP, cyan for Cu_0.1_BCP and light military green for Cu_0.2_BCP (Fig. 1D). Their SSA values were within a close range: 2.0 ± 0.1 m^2^/g, 1.8 ± 0.1 m^2^/g, and 2.3 ± 0.1 m^2^/g, respectively. Powder analysis by ICP/AES confirmed the copper(II) doping values in the final compositions of calcined powders and revealed an increase in copper(II) release into the culture medium (Table 1).

Equivalent characterization methods were used to analyze the BCP powders synthesized *via* the sol-gel method, and comparable physicochemical features were observed (Fig. S1 and Table S1); only carbonate traces were detected (Fig. S1B) in the powders prepared by the sol-gel method [40].

### 3.2 Effect of copper(II) ions-doped BCP powders on cytokine production

**Fig. 2:**
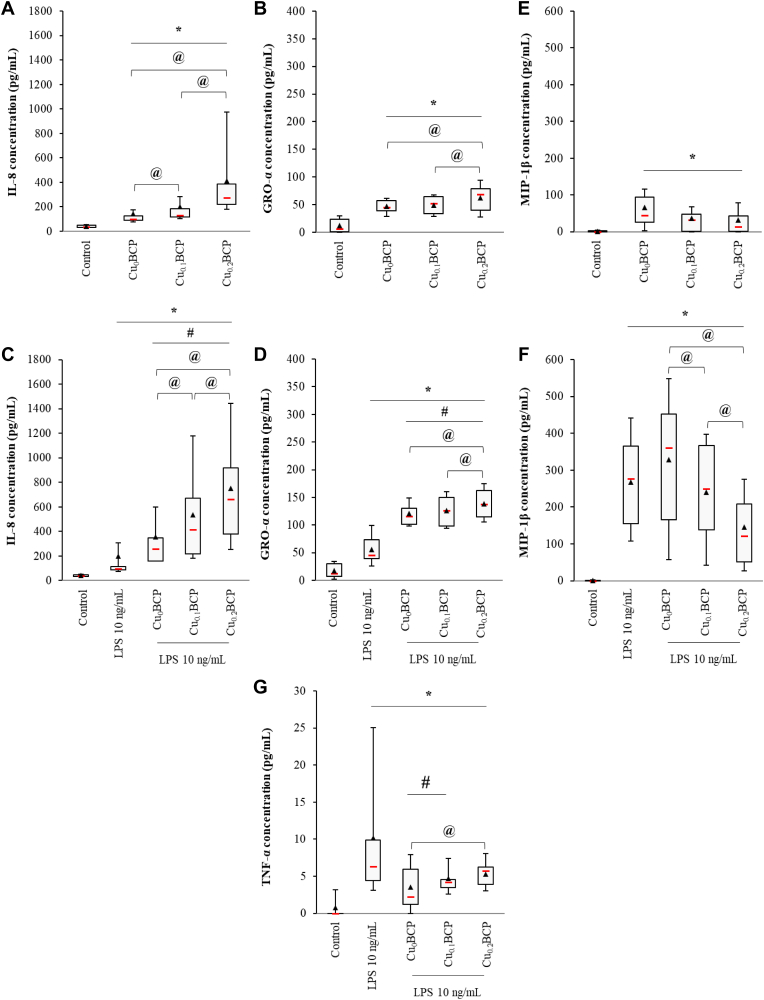
Copper (II) ions-doped BCP powders increased IL-8 and GRO-α, but not MIP-1β and TNF-α secretion by PMNs. (**A, B, E**) ELISA measurements of the IL-8, GRO-α, MIP-1β concentrations in unexposed and BCP powder-exposed PMNs and (**C, D, F**) LPS-stimulated PMN supernatants after 4 h. (**G**) ELISA measurements of TNF-α concentrations in the supernatants of unexposed and BCP powder-exposed PMNs stimulated by LPS after 4 h *p < 0.05 compared with unexposed (control) cells. **#***p* < 0.05 compared with unexposed LPS-stimulated PMNs. **@***p* < 0.05 between the indicated conditions. n = 9–11.

To evaluate the impact of Cu-doped BCP materials on acute inflammation, the concentrations of three chemokines and one pro-inflammatory cytokine secreted by PMNs and LPS-stimulated PMNs in contact with the powders were studied by ELISA. IL-8 and GRO-α are two chemokines abundantly produced by PMNs exerting stimulatory and chemotactic activities toward neutrophils themselves, whereas MIP-1β is predominantly chemotactic for monocytes and macrophages. Without costimulation, an increase in the amount of IL-8 and GRO-α secreted by PMNs in contact with all powders was noticed compared to the control, with a significant copper(II) concentration-dependent effect (*p* < 0.05) (Fig. 2A and B). Upon LPS costimulation, IL-8 and GRO-α production by PMNs was further increased 2- to 3-fold (Fig. 2C and D). Copper(II) doping appears to strongly increase IL-8 and, in a lesser extent, GRO-α production by neutrophils compared to the copper-free control materials and LPS alone; a concentration-dependent effect was also noticed. All BCP powders, with or without LPS costimulation, triggered a significant MIP-1β production by PMNs compared to control (*p* < 0.05) (Fig. 2E and F). Of importance, upon LPS costimulation we observed a copper(II) concentration-dependent reduction in MIP-1β secretion compared to the copper-free control material (Fig. 2F). TNF-α is a proinflammatory cytokine neosynthesized by PMNs in response to a proinflammatory stimulus and is known to activate inflammatory cells, including monocytes and macrophages. Under the control condition, no TNF-α secretion was detected. Moreover, the BCP materials alone do not induce the production of TNF-α by neutrophils (data not shown). TNF-α production was measured following LPS stimulation at a low median value of 7.5 pg/mL (Fig. 2G). Costimulation with LPS and the BCP powders did not upregulate TNF-α production. We noted that Cu_0_BCP and Cu_0.1_BCP decreased the TNF-α concentration in the conditioned supernatants (*p* < 0.05), but Cu_0.2_BCP restored TNF-α secretion compared to LPS (*p* > 0.05). Similar trends were observed for IL-8, GRO-α, and MIP-1β when using sol-gel-based BCP powders, whereas there was no variation with LPS costimulation for TNF-α (Fig. S2).

### 3.3 Effect of copper(II) ions-doped BCP powders on MMP9-related gelatinolytic activity

**Fig. 3:**
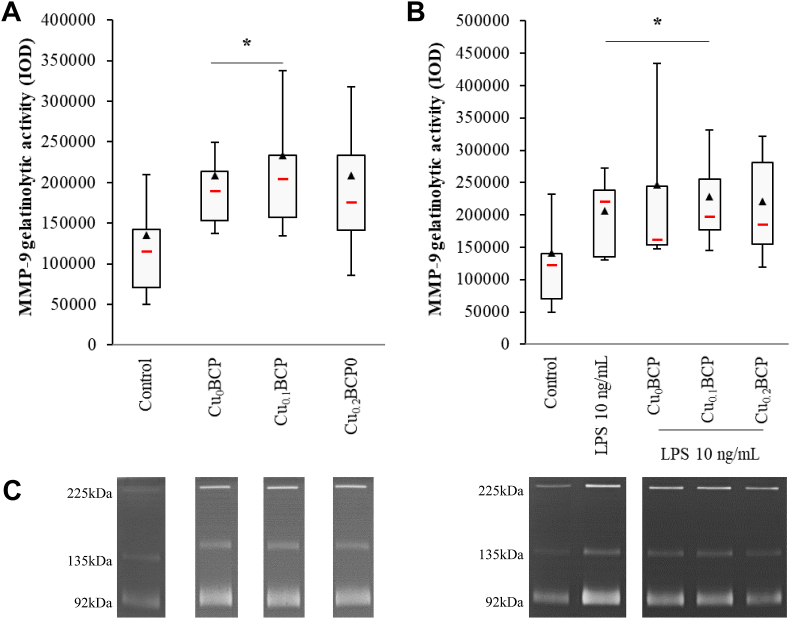
Copper (II) ions-doped BCP powders did not potentiate MMP-9-related gelatinolytic activity. (**A**) Total MMP-9 gelatinolytic activity in unexposed and BCP powder-exposed PMNs and (**B**) LPS-stimulated PMN supernatants after 4 h. (**C**) Zymography gels from representative donors. *p < 0.05 *vs.* control. n = 9–10.

The influence of the copper(II)ions -doped BCP samples on MMP-9-related gelatinolytic activity in PMN-conditioned supernatants was assessed by gelatin zymography. The Cu_0_BCP and Cu_0.1_BCP powders induced significant increases in MMP-9-related gelatinolytic activity (1.8-fold compared with the control, *p* < 0.05) (Fig. 3A). In the presence of LPS, a similar pattern of MMP-9 activity was observed, with a significant increase for Cu_0_BCP and Cu_0.1_BCP compared with the control condition (Fig. 3B). Only an upward trend was observed with the materials synthesized *via* the sol-gel route (Fig. S3).

### 3.4 NET formation

**Fig. 4:**
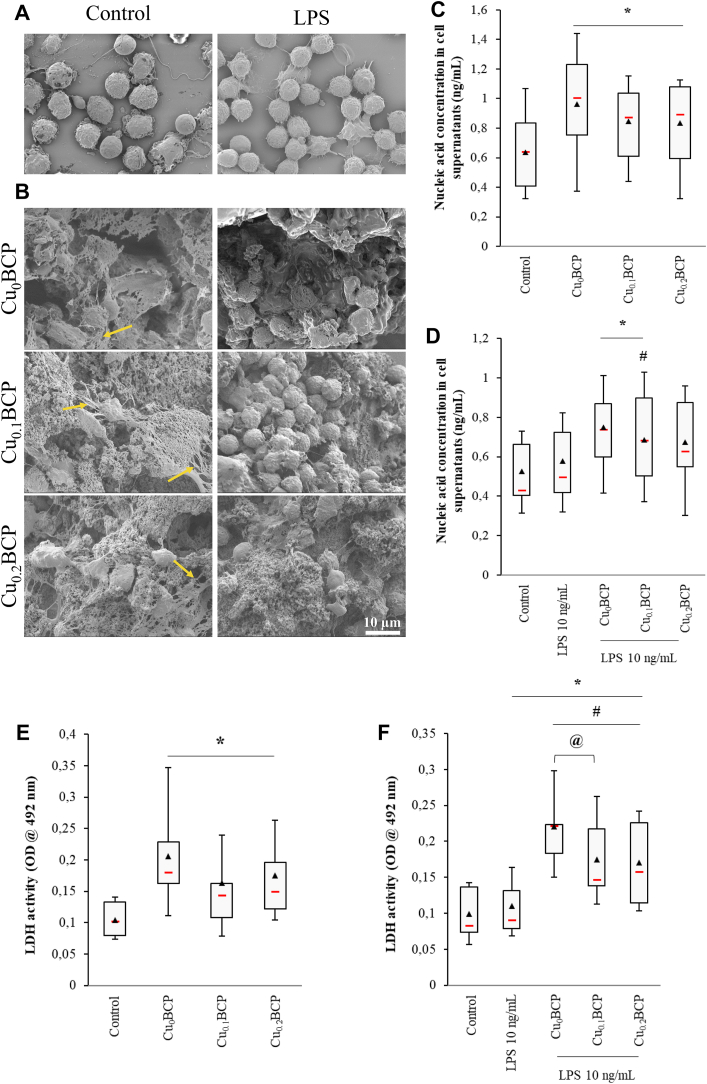
Copper (II) ions-doped BCP powders did not induce necrotic cell death. (**A** and **B**) SEM images of PMNs alone and in the presence of BCP powders without or with LPS stimulation (2500 × , scale bar = 10 μm) (one representative donor, n = 4). (**C**) Nucleic acid concentration in unexposed and BCP powder-exposed PMNs and (**D**) LPS-stimulated PMN supernatants after 4 h. (**E**) LDH activity in unexposed and BCP powder-exposed PMNs and (**F**) LPS-stimulated PMN supernatants after 4 h *p < 0.05 *vs.* control. #*p* < 0.05 *vs.* LPS. @ *p* < 0.05 between the indicated conditions. n = 9–11.

SEM allowed us to observe the presence of NETs in contact with the different BCP powders. No or very few NETs were observed under LPS costimulation conditions (Fig. 4A and B). Quantification of the soluble fraction of these nucleic acids shows that a larger quantity of NETs was produced in the presence of powders compared to the control (Fig. 4C). Under LPS-costimulated conditions, only Cu_0_BCP and Cu_0.1_BCP showed a slight but significant increase in NET formation (*p* < 0.05) (Fig. 4D). Using the materials synthesized *via* the sol-gel route, no significant variation was observed (Figs. S4A–S4D).

### 3.5 LDH measurement

Cell mortality was evaluated *via* LDH measurements in neutrophil supernatants after 4 h of culture with or without LPS costimulation. Powders induced a significant increase in the activity of this enzyme in comparison with the control condition (1.9-fold, *p* < 0.05) (Fig. 4E). In LPS-stimulated PMN-conditioned supernatants, LDH activity was significantly increased with and without the BCP powders (*p* < 0.05 compared with the control) (Fig. 4F). However, in this proinflammatory environment, the copper(II) ions-doped powders tended to decrease LDH release, but this effect was significant for only the Cu_0.1_BCP powder (decrease of 32% compared to the Cu_0_BCP powder). Sol-gel-based powders did not induce appreciable changes in LDH release despite a similar trend as described above (Figs. S4E and S4F).

### 3.6 Effect of copper(II) ions-doped BCP powders on the *in vivo* recruitment of PMNs

**Fig. 5:**
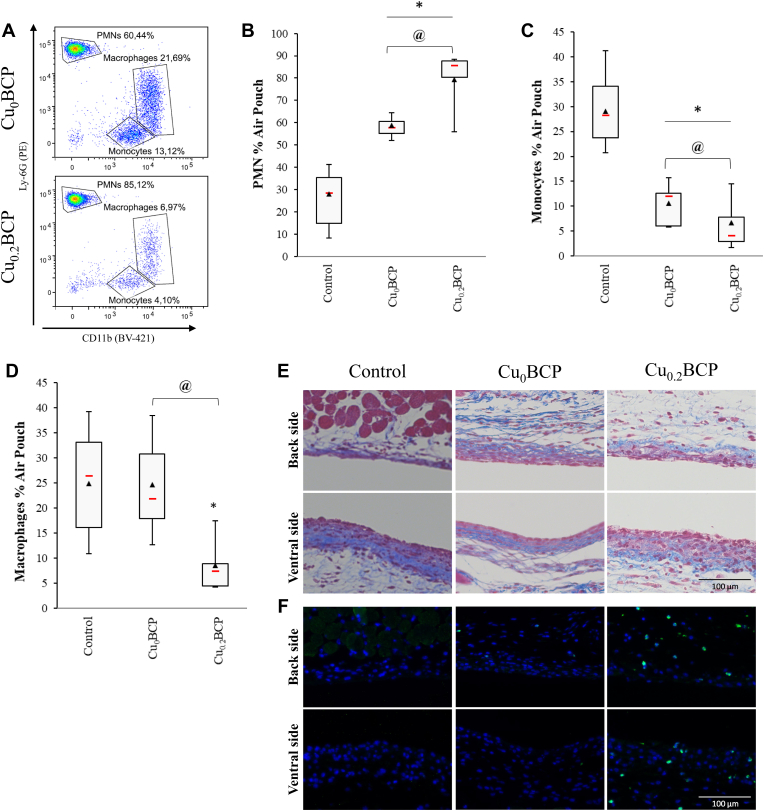
Copper (II) ions-doped BCP powders favored neutrophil recruitment *in vivo*. (**A**) Representative cytograms of the air pouch cell population. (**B** to **D**) Quantification of PMNs, monocytes and macrophages recruited in the mouse air pouches 6 h after injection of DPBS/DMSO (control) or BCP powders determined by flow cytometry. *p < 0.05 *vs.* control. @ *p* < 0.05 between the indicated conditions. n = 10 (for each powder condition) and n = 16 (for the control condition). (**E**) Representative image of Masson's trichrome-stained histological sections and (**F**) immunofluorescence staining (nuclei in blue and Ly6G-positive PMNs in green) of the air pouch membrane 6 h after injection of DPBS/DMSO (control) or BCP powders in suspension. Scale bar = 100 μm. (For interpretation of the references to colour in this figure legend, the reader is referred to the Web version of this article.)

**Fig. 6:**
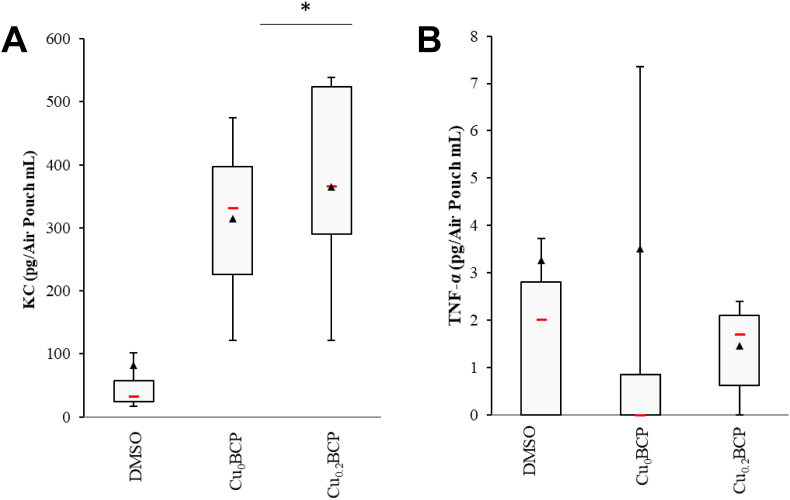
BCP powders increased the KC but not the TNF-α concentration in air pouches. (**A**) ELISA measurements of KC and (**B**) TNF-α concentrations in the mouse air pouch 6 h after injection of DPBS/DMSO (control), 10 ng/mL LPS or BCP powders. *p < 0.05 *vs.* control. **#***p* < 0.05 *vs.* LPS. @ *p* < 0.05 between the indicated conditions. n = 10 to 16.

To obtain further insights into the ability of the BCP powders to modulate leukocyte recruitment *in vivo,* we used the murine air pouch model of tissue inflammation. The total number of leukocytes in the exudate was counted, and PMNs, monocytes and macrophages were quantified by flow cytometry. In this model, all powders increased the recruitment of total leukocytes compared to the control (*p* < 0.05; data not shown). An increase the percent of PMNs (representing 57%–85% of the total leukocytes in the median) in the air pouch to the detriment of monocytes (representing 4%–12% of total leukocytes in median) and macrophages (representing 6%–21% of total leukocytes in median; *p* < 0.05) was found (Fig. 5A–D). Moreover, the recruitment of PMNs increased with Cu_0.2_BCP compared to the Cu-free control, whereas monocyte and macrophage percentages decreased significantly. The histological images also exhibited an increased neutrophil presence inside the air pouch tissue in the presence of copper(II) ions (Fig. 5E and F). Neutrophil recruitment matched with the global increase in KC concentration in the air pouches containing BCP powders, with a trend toward a more sustained KC concentration for the Cu_0.2_BCP powder (Fig. 6A). However, these powders did not induce noticeable TNF-α production *in vivo* (Fig. 6B). Of interest, sol-gel-based powders exhibited similar biological responses *in vivo* inside the air pouch regardless of the presence of copper(II) ions (Figs. S5A–S5D). Nevertheless, histological section analyses exhibited figures similar to those observed with aqueous precipitation-based powders (Fig. 5E and F). The KC concentration in the air pouch exudate increased compared to the control condition only in the absence of copper(II) ions, and the TNF-α concentration remained stable in the presence of the BCP powders (Fig. S6).

## 4 Discussion

The main purpose of this study was to investigate the potential effect of copper(II) ions doping on the acute inflammatory response of Cu_z_BCP-based biomaterials, with z = 0, 0.1 and 0.2. To this aim, the secretion of proinflammatory cytokines by neutrophils, especially IL-8, GRO-α, MIP-1β, and TNF-α, was investigated. IL-8 and GRO-α are two CXC-chemokines with neutrophil-activating properties produced by various types of cells [41,42]. Both chemokines play a causative role in acute inflammation by recruiting, activating, and facilitating PMN transmigration through the endothelium [[43], [44], [45]]. MIP-1β is a CC-chemokine which acts as a potent chemotactic/activating factor for monocytes, macrophages, and lymphocytes [45]. TNF-α is known as a proinflammatory cytokine, enabling leukocyte activation and adhesion, and increasing ROS production. None of the BCP powders presented here had an intrinsic inflammatory effect. Indeed, no production of TNF-α was detected in the PMN culture supernatants after contact with BCP powders. On the other hand, all of these powders induced a slight increase in the release of IL-8, GRO-α, and MIP-1β by neutrophils. Of interest, copper(II) ion doping increased both CXC-chemokines (IL-8, GRO-α) and conversely tend to decrease CC-chemokine (MIP-1β). This chemokine production profile would strengthen the recruitment of additional immune cells, especially neutrophils, without necessarily overactivating them. As copper(II) ions is known for its antimicrobial properties, the use of copper(II) ions-doped biomaterials could be particularly relevant in surgical sites with septic risk. To mimic an inflammatory site in a septic environment, PMNs were costimulated with both BCP-based powders and LPS. The IL-8 and GRO-α production observed during cell stimulation by the copper(II) ions-doped powders increased in the presence of LPS. The MIP-1β production also increased after PMN costimulation with undoped-BCP powders and LPS. Interestingly, copper(II) ions doping significantly reduced MIP-1β production suggesting a moderate monocyte/macrophage recruitment by PMNs in an inflammatory context. We have thus evidenced differential regulation of our three chemokines under the effect of copper(II) doping. To date no data may explain such an observation and this will deserve further investigations to be understood. In contrast, the cumulative effect of LPS and doped or undoped BCP powders did not increase TNF-α secretion. It was established that Toll-like receptor 4 (TLR4), a pattern recognition receptor involved in the detection of LPS by leukocytes, is required for the production of TNF-α induced by CaP-based particles on macrophages [46]. TLR4 is also present on the surface of neutrophils [47]. As LPS has been widely described to activate the TLR4 signaling pathway in a variety of cell types [48], it can be assumed that LPS-bound TLR4 would no longer be available for binding CaP-based particles. This may be part of the explanation for the absence of a cumulative effect between the BCP powders and LPS under our costimulation conditions. In an inflammatory context, the BCP samples moderated TNF-α production, while copper(II) ions doping seemed to ensure a minimum level of mediator production, keeping the activation of immune cells at a moderate level. Furthermore, the study of the gelatinolytic activity of MMP-9, the main protease secreted by PMNs through the release of tertiary granules [49] that participates in tissue degradation during exacerbated inflammation, showed basal production by PMNs in the absence of LPS, which was only moderately increased with the BCP powders. The copper(II) ions-doped materials tended to inhibit this increase under LPS stimulation. MMP-9 is involved in the basement membrane remodeling of blood vessels during the early inflammatory response, facilitating the transendothelial migration of leucocytes [50,51]. Several nonmatrix substrates can also be cleaved by MMP-9, including IL-8, and cleavage of IL-8 by MMP-9 potentiates its chemoattractant effect [49]. It is furthermore interesting that the secretion of MMP-9 by PMNs is linked to the binding of IL-8 to its receptors. This evidence suggests that uncontrolled MMP-9-associated gelatinolytic activity may lead to persistent inflammation. Here, copper(II) ions doping appeared to not increase MMP-9 activity induced by the BCP powders, as we noticed a trend toward normalization of MMP-9 activity with the Cu_0.2_BCP powder.

During their activation, neutrophils are likely to produce NETs, the extracellular structure of decondensed chromatin that facilitates the immobilization and subsequent destruction of microorganisms. The BCP powders displayed a basal capacity to induce NET formation, which was slightly altered in the presence of LPS. An increase in the soluble nucleic acid concentration was noticed in the conditioned supernatants with the powders synthesized *via* the aqueous precipitation method, suggesting more important NET formation. In the literature, a distinction is made between the formation of NETs from mitochondrial DNA (along with proteins enabling the rapid resolution of inflammation) and NETosis, a distinct program of necrotic cell death involving the release of all cell contents [52]. To elucidate the mechanism involved in the contact with BCP samples, we simultaneously studied LDH activity. LDH is a cytoplasmic enzyme present in all cells that is released into culture supernatants following damage to the plasma membrane. The powders visibly induced a slight increase in LDH activity, which, compiled with the nucleic acid quantification data, supports NETosis of PMNs in contact with powders synthesized *via* the aqueous precipitation method. Nevertheless, it should be noted that the increase in LDH activity remained far below the LDH activity signal of cell death control (OD_492nm_ = 1.2, data not shown), highlighting the low cytotoxicity of the materials. Finally, the effect of the copper(II) ions-doped BCP materials on inflammatory cell recruitment was evaluated in a murine model of acute inflammation. *In vivo*, the BCP materials induced an increase in the number of leukocytes recruited into the air pouch, especially neutrophils. This result is congruent with the above-discussed increase in PMN IL-8 secretion *in vitro* and increased KC concentration measured in the air pouch exudate *in vivo* for the powder produced *via* aqueous precipitation, whereas the decreased KC concentration measured with sol-gel route-synthesized Cu_0.2_BCP led to no variation in leukocyte population equilibrium. Copper(II) ions-doped BCP materials potentiated PMN recruitment compared to undoped samples, whereas monocyte/macrophage recruitment was decreased.

Cation doping in HA and/or TCP lattices generally resulted in a pronounced inhibition of the inflammatory response. For instance, Zn-substituted HA succeeded in decreasing IL-8 production by neutrophils and lowering TNF-α and IL-1β production from monocytes/macrophages [17,46], and Sr-substituted HA has shown an anti-inflammatory effect on monocytes/macrophages [18]. In association with calcium phosphates, it has been shown that magnesium switch macrophages to a M2 anti-inflammatory phenotype [53]. In an infectious context, reducing the functional capacities of immune cells can be devasting, as it works in favor of microorganism progression.

This work demonstrated that copper(II) ions doping in BCP biomaterials could potentiate the recruitment of PMNs, maintain the rate of inflammatory cell activation during the implantation of the material, and thus succeed in containing the proteolytic risk generally associated with osteolysis phenomena. It can be assumed that the observed biological effects would be directly related to the release of copper(II) ions into the environment. Combined with the data on the nontoxic, antibacterial and angiogenic properties of these Cu-doped CaP biomaterials [22, 54], these results support the hypothesis that copper(II) ions doping can endow BCP materials with new perspectives in bone replacement and regeneration.

## Credit author statement

Thoraval Léa: Conceptualization, Formal analysis, Investigation, Methodology, Validation, Visualization, Writing original, Reviewing editing. Thiébault Emilie: Formal analysis, Investigation, Reviewing editing. Siboni Renaud: Formal analysis, Investigation, Reviewing editing. Moniot Aurélie: Formal analysis, Investigation, Reviewing editing. Guillaume Christine: Formal analysis, Investigation, Reviewing editing. Jacobs Aurélie: Formal analysis, Investigation, Resources, Reviewing editing. Nedelec Jean-Marie: Conceptualization, Formal analysis, Resources, Supervision, Reviewing editing. Renaudin Guillaume: Formal analysis, Project administration, Resources, Reviewing editing. Descamps Stéphane: Formal analysis, Resources, Reviewing editing. Valfort Olivier: Formal analysis, Investigation, Resources, Reviewing editing. Gangloff Sophie C: Funding acquisition, Writing original, Reviewing editing. Braux Julien: Formal analysis, Methodology, Validation, Reviewing editing. Marchat David: Conceptualization, Formal analysis, Investigation, Resources, Supervision, Validation, Visualization, Writing original, Reviewing editing. Velard Frédéric: Conceptualization, Formal analysis, Funding acquisition, Project administration, Supervision, Validation, Writing original, Reviewing editing.

## Declaration of competing interest

The authors declare that they have no known competing financial interests or personal relationships that could have appeared to influence the work reported in this paper.

## Data Availability

Data will be made available on request.
