# Supplementary figures and images for: The acute inflammatory response to copper(II)-doped biphasic calcium phosphates
Source: Mater Today Bio. 2023 Oct 4;23:100814. doi: 10.1016/j.mtbio.2023.100814 (PMC10568289; doi:10.1016/j.mtbio.2023.100814)

## Slide 1
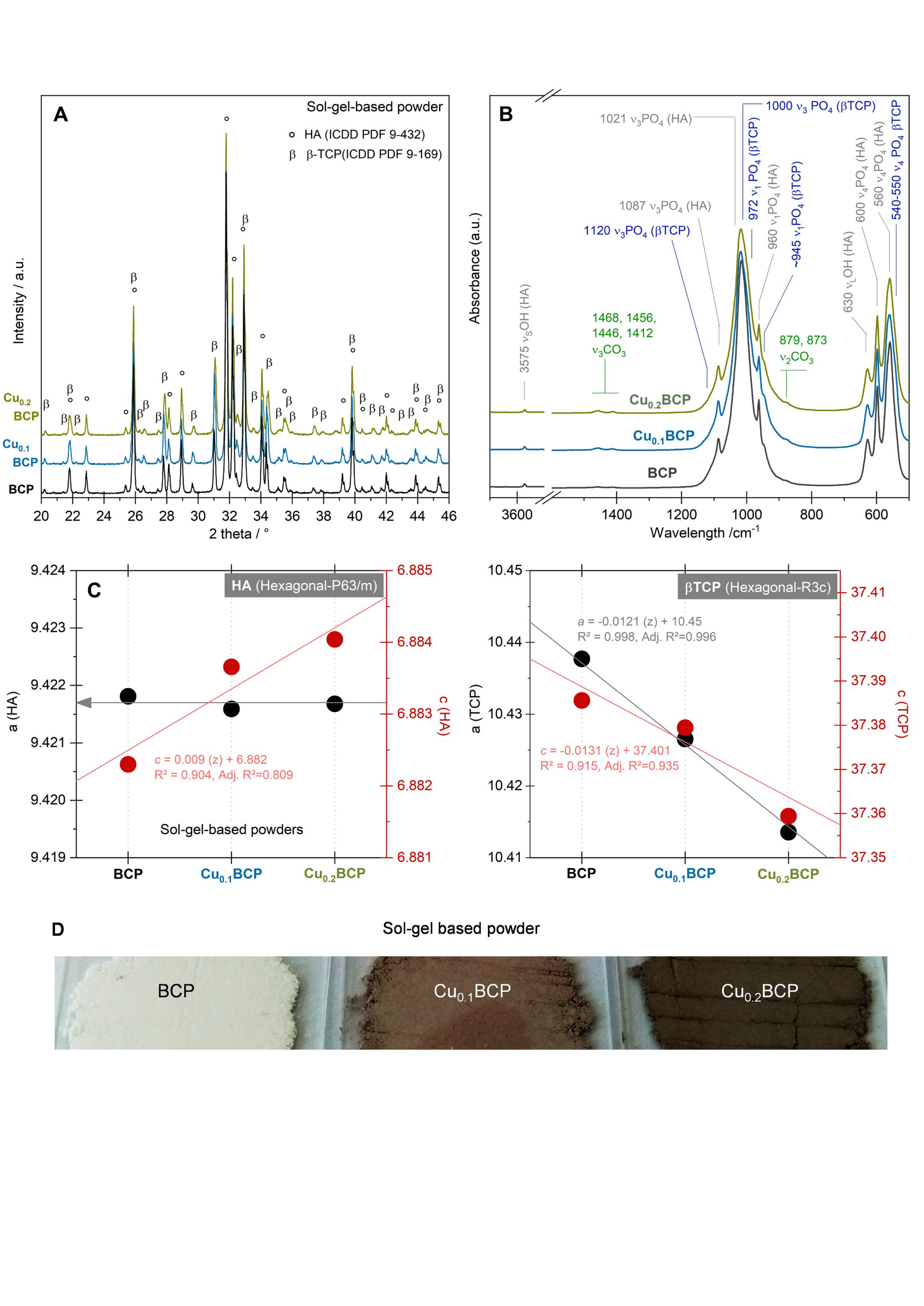

Supplement: Multimedia component 1 [file mmc1.pptx]

## Slide 1
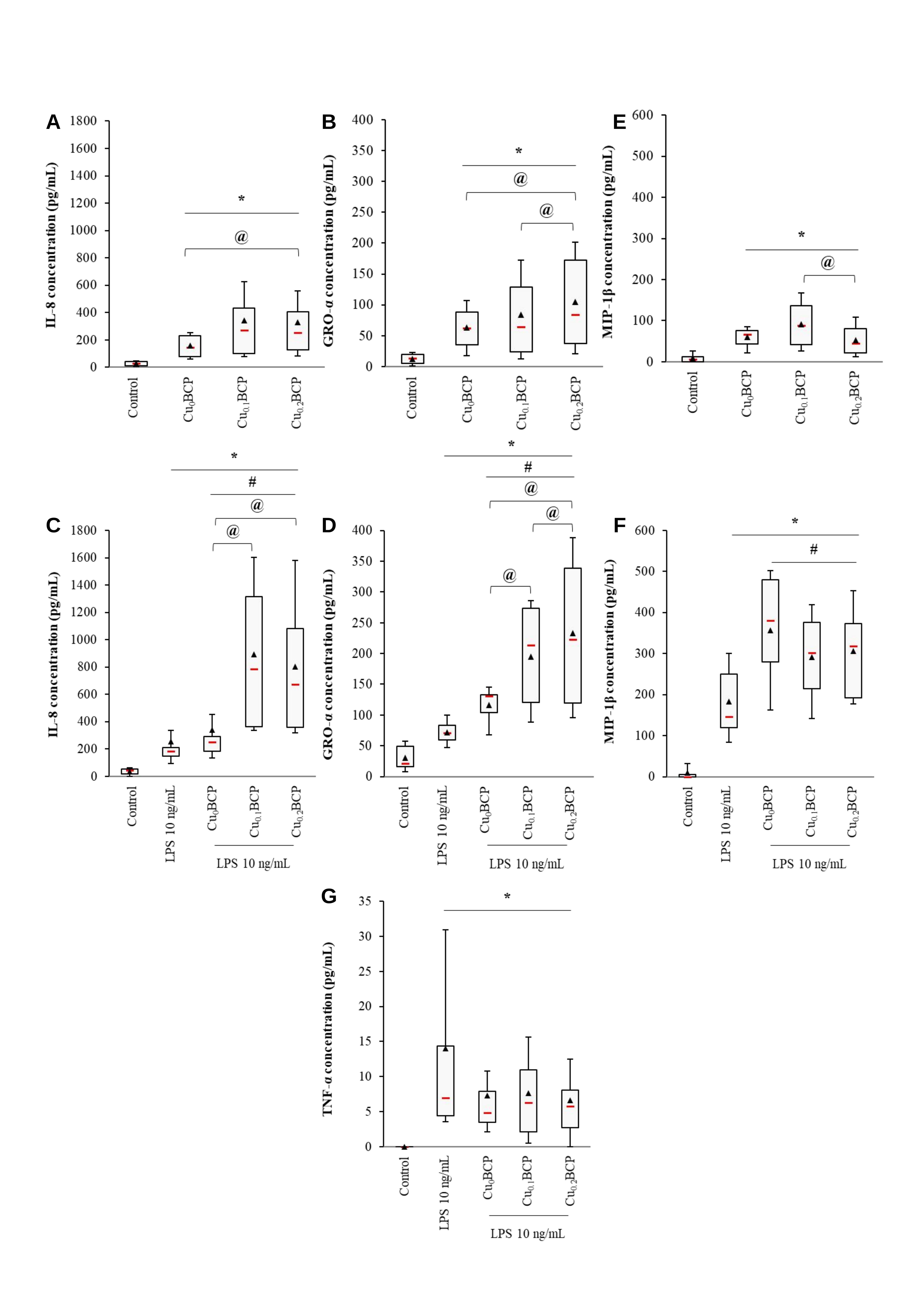

A
B
E
C
D
F
G

Supplement: Multimedia component 2 [file mmc2.pptx]

## Slide 1
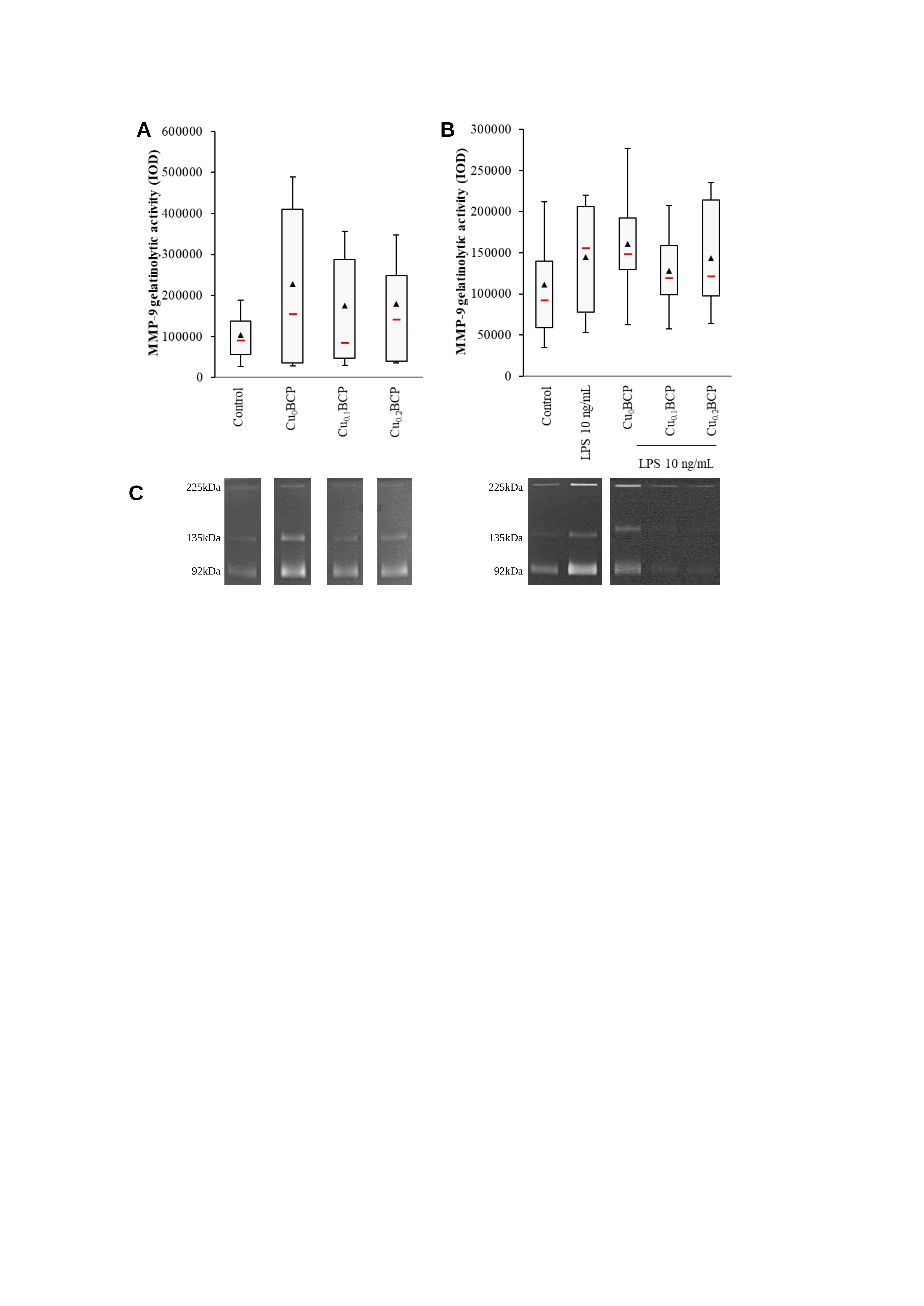

A
B
C
225kDa
225kDa
135kDa
135kDa
92kDa
92kDa

Supplement: Multimedia component 3 [file mmc3.pptx]

## Slide 1
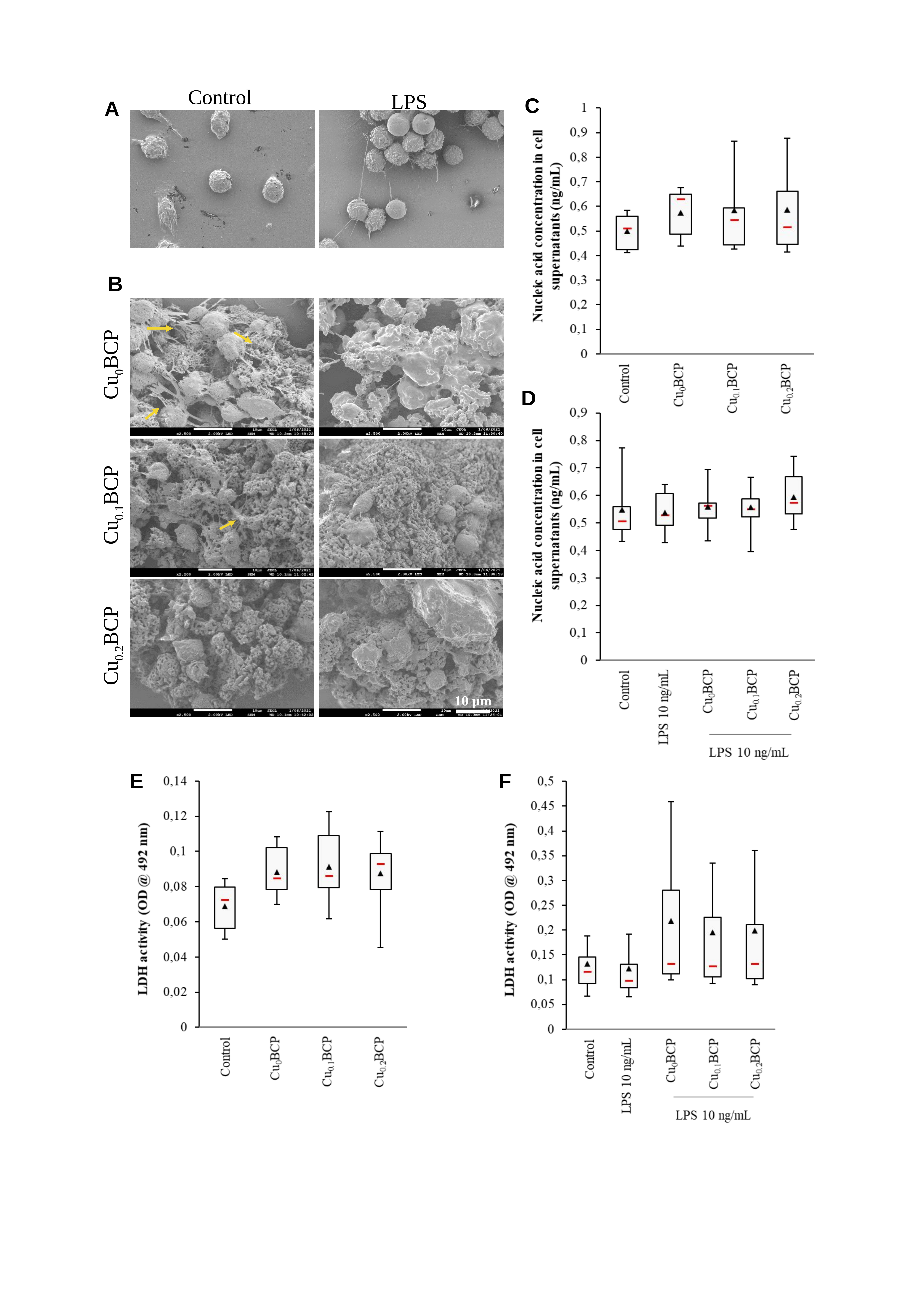

Control
LPS
C
A
B
Cu0BCP
D
Cu0.1BCP
Cu0.2BCP
10 µm
E
F

Supplement: Multimedia component 4 [file mmc4.pptx]

## Slide 1
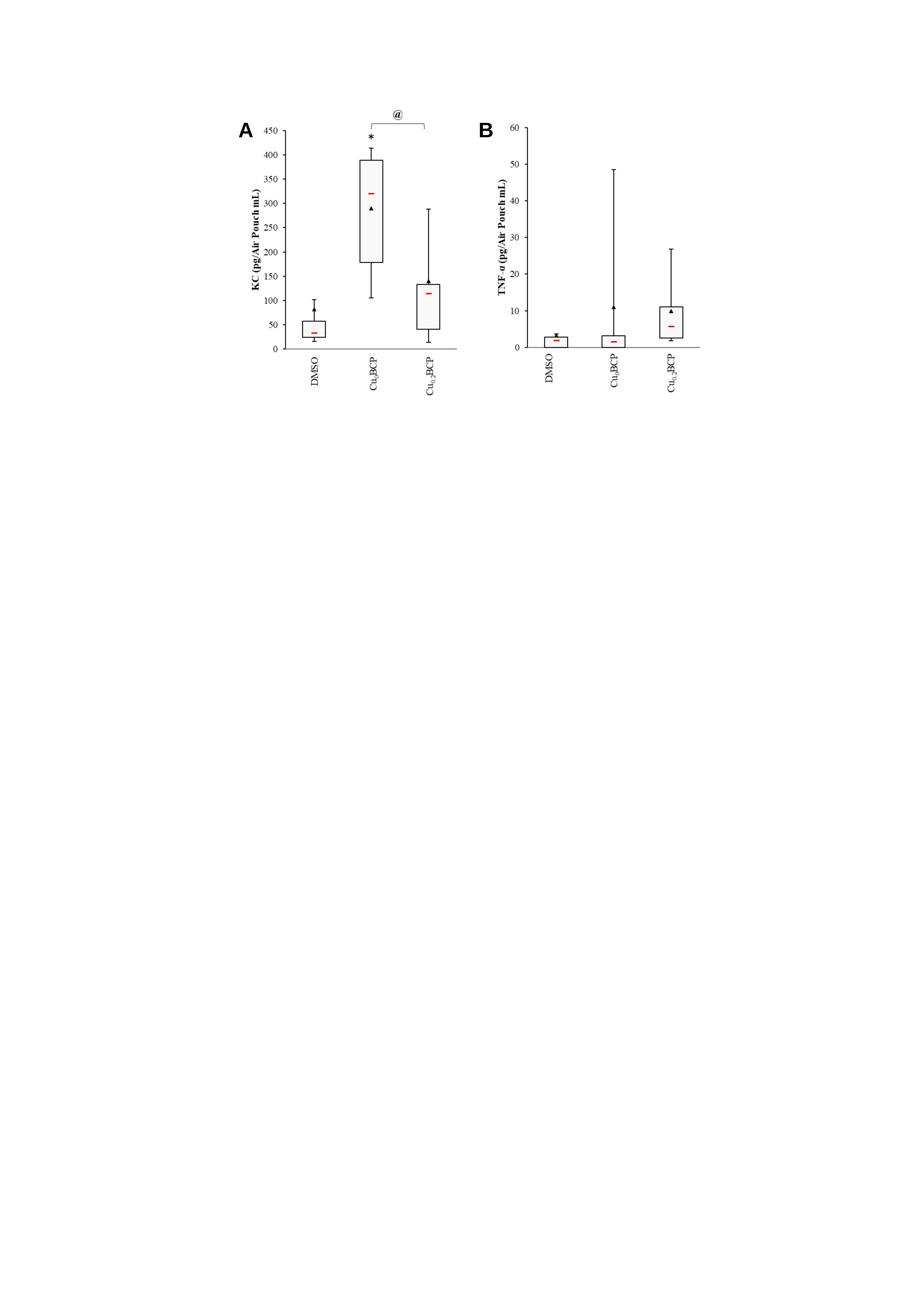

A
B

Supplement: Multimedia component 6 [file mmc6.pptx]
