# Supplementary material for: The acute inflammatory response to copper(II)-doped biphasic calcium phosphates
Source: Mater Today Bio. 2023 Oct 4;23:100814. doi: 10.1016/j.mtbio.2023.100814 (PMC10568289; doi:10.1016/j.mtbio.2023.100814)
Supplement: Multimedia component 5 [file mmc5.pptx]

## Slide 1
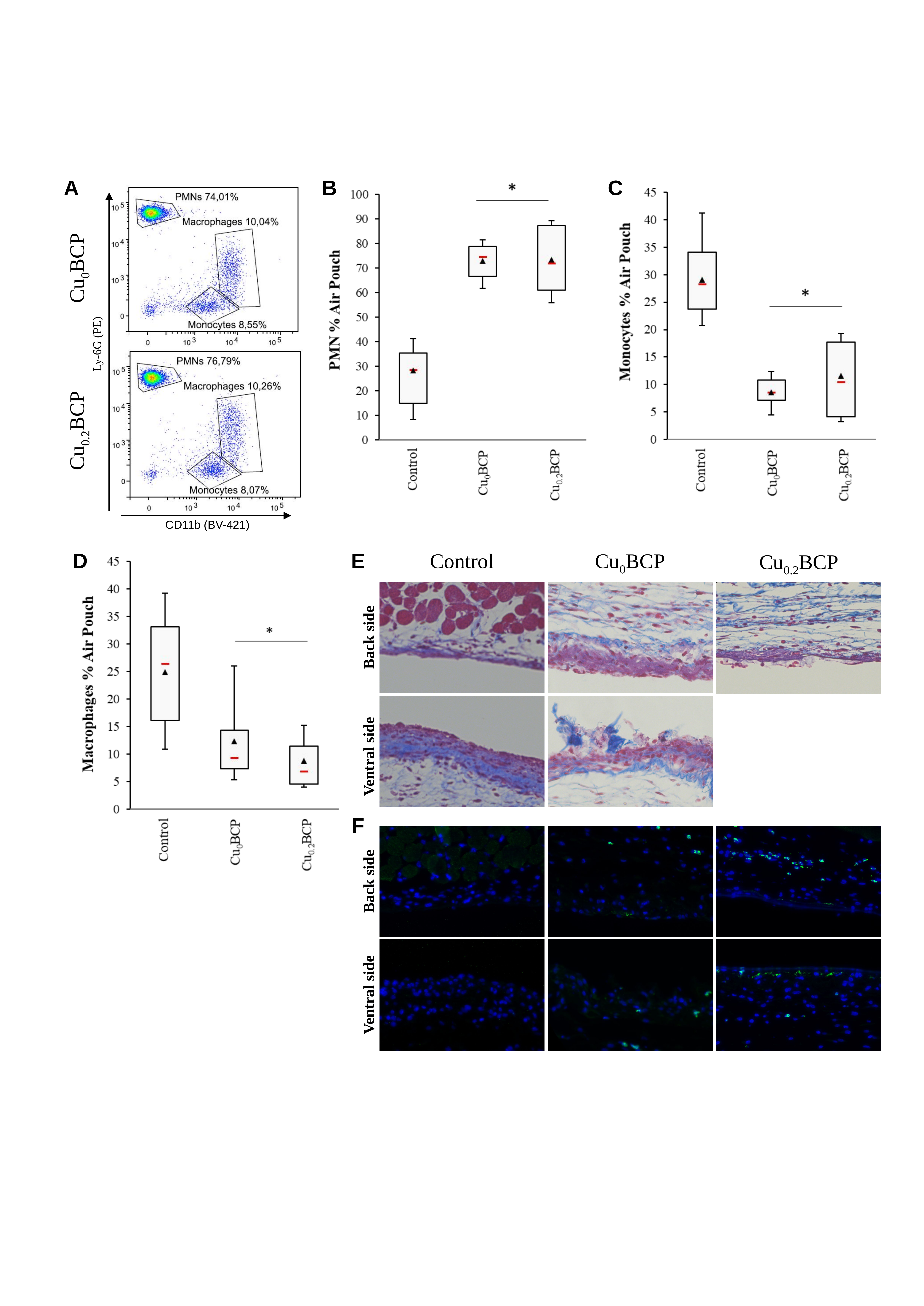

A
B
C
Cu0BCP
Ly-6G (PE)
Cu0.2BCP
CD11b (BV-421)
D
Cu0BCP
E
Control
Cu0.2BCP
Back side
Ventral side
F
Back side
Ventral side
