# Supplementary material for: The acute inflammatory response to copper(II)-doped biphasic calcium phosphates
Source: Mater Today Bio. 2023 Oct 4;23:100814. doi: 10.1016/j.mtbio.2023.100814 (PMC10568289; doi:10.1016/j.mtbio.2023.100814)
Supplement: Multimedia component 7 [file mmc7.docx]

Table S1. Parameters of the synthesis of the sol-gel-based powders, their final compositions after heat treatment at 900 °C for 15 h under air, and the Ca, Cu and P concentrations in RPMI 1640 Glutamax^®^ medium incubated for 4 h at 37 °C with the Cu_z_BCP powders (1.7 mg/mL).

| Sample name | Chemical synthesis: Reagent amounts | | | | Final calcined powder composition | | | | Powder dissolution: Element concentration in RPMI media | | |
| --- | --- | --- | --- | --- | --- | --- | --- | --- | --- | --- | --- |
|  | z | n_Ca_ | n_P_ | n_Cu_ | HA/β-TCP mass ratio | Ca/P | x | Cu/Ca ^b^ | Ca | Cu | P |
|  | - | / 10^-3^ mol | | | From  Rietveld  and [35] | from Eq. 4 | Eq. 4 | by ICP/AES | / mol/L |  |  |
| Cu_0_BCP | 0 | 19.9 | 5.9 | 0 | 78±6/22±6 | 1.628  ±0.010 | 0.23 | 0 | 0.59  ±0.04 | 0.00  ±0.00 | 5.29  ±0.07 |
| Cu_0.1_BCP | 0.1 | 19.9 | 5.9 | 0.2 | 70±5/30±5 | 1.614  ±0.008 | 0.32 | 0.010  ±0.001 | 0.58  ±0.01 | 0.12  ±0.01 | 5.27  ±0.06 |
| Cu_0.2_BCP | 0.2 | 19.9 | 5.9 | 0.4 | 71±5/29±5 | 1.616  ±0.009 | 0.31 | 0.020  ±0.001 | 0.57  ±0.01 | 0.21  ±0.01 | 5.22  ±0.05 |
